# Genome-Wide Investigation of Class III Peroxidase Genes in *Brassica napus* Reveals Their Responsiveness to Abiotic Stresses

**DOI:** 10.3390/plants13070942

**Published:** 2024-03-25

**Authors:** Obaid Ullah Shah, Latif Ullah Khan, Sana Basharat, Lingling Zhou, Muhammad Ikram, Jiantao Peng, Wasi Ullah Khan, Pingwu Liu, Muhammad Waseem

**Affiliations:** 1 School of Breeding and Multiplication (Sanya Institute of Breeding and Multiplication), College of Tropical Agriculture and Forestry, Hainan University, Sanya 572025, China; obaidus890@gmail.com (O.U.S.); latif.hainu3103@gmail.com (L.U.K.); m15237352151@163.com (L.Z.); drikram@gzhu.edu.cn (M.I.); jiantaopeng@hainanu.edu.cn (J.P.); wasibiotechnologist@gmail.com (W.U.K.); 2 Department of Botany, University of Agriculture Faisalabad, Faisalabad 38000, Pakistan; sanabasharat@gmail.com

**Keywords:** peroxidases, expression pattern, abiotic stress, rapeseed

## Abstract

*Brassica napus* (*B. napus*) is susceptible to multiple abiotic stresses that can affect plant growth and development, ultimately reducing crop yields. In the past, many genes that provide tolerance to abiotic stresses have been identified and characterized. Peroxidase (POD) proteins, members of the oxidoreductase enzyme family, play a critical role in protecting plants against abiotic stresses. This study demonstrated a comprehensive investigation of the *POD* gene family in *B. napus*. As a result, a total of 109 *POD* genes were identified across the 19 chromosomes and classified into five distinct subgroups. Further, 44 duplicate events were identified; of these, two gene pairs were tandem and 42 were segmental. Synteny analysis revealed that segmental duplication was more prominent than tandem duplication among *POD* genes. Expression pattern analysis based on the RNA-seq data of *B. napus* indicated that *BnPOD* genes were expressed differently in various tissues; most of them were expressed in roots rather than in other tissues. To validate these findings, we performed RT-qPCR analysis on ten genes; these genes showed various expression levels under abiotic stresses. Our findings not only furnish valuable insights into the evolutionary dynamics of the *BnPOD* gene family but also serve as a foundation for subsequent investigations into the functional roles of *POD* genes in *B. napus*.

## 1. Introduction

Peroxidases are a large family of ubiquitous isozymes that play an important role in plant growth, development, and defense mechanisms [1] and have been categorized into hemoglobin peroxidases and non-hemoglobin peroxidases [2,3]. Non-hemoglobin peroxidases include (i) class I peroxidases, which are mainly involved in oxidative stress tolerance [4]; (ii) class II peroxidases, which are found in fungi and play an essential role in lignin metabolism [5]; and (iii) class III peroxidases, which are a plant-specific ubiquitous multigenic family and play pivotal roles in plant growth, development, and stress responses [6,7]. POD proteins have highly conserved amino acid residues essential for glycosylation and cysteine–histidine catalytic reactions [7] and are involved in different physiological and developmental processes, including cell wall metabolism, lignification, auxin metabolism, and tolerance and defense against biotic and abiotic stresses [8,9,10].

Previously, many functional studies have identified the role of PODs in the tolerance of crop plants to different environmental stresses [11,12,13]. For example, the overexpression of AtPrx64 is involved in aluminum tolerance in *Nicotiana tabacum* [11], whereas the overexpression of the cold-inducible peroxidase gene (RCI3) in Arabidopsis shows increases in dehydration and salt tolerances [12]. Similarly, the CrPrx and CrPrx1 genes from Catharanthus roseus are involved in germination and exhibit better cold tolerance in tobacco [13], while a mutant of TaPRX-2A in Triticum aestivum is involved in salinity tolerance [14]. In RNA-seq and microarrays, the expression profiles of several peroxidase (PRX) genes showed differential expressions under different stresses in wheat and maize, indicating the role of peroxidase in response to abiotic stresses [15,16]. These findings suggested that plant PODs are anticipated to be involved in abiotic stresses in multiple crop plants.

In recent years, genome-scale bioinformatics analyses of multigenic families, like peroxidases, have been greatly helpful in understanding the physiological roles and characteristics of these gene families. The *POD* gene family has been reported in numerous plant species, including *Arabidopsis thaliana* [17], *Manihot esculenta* [18], *Pyrus pyrifolia* [19], *Zea mays* [16], *Oryza sativa* [7], *Citrus sinensis* [20], *Vitis vinifera* [21], *Glycine max* [22], and *Capsicum annuum* L. [23]. Genetic evidence has indicated that *POD* serves as a family of enzymes responsive to abiotic stresses in diverse plant species [24].

*Brassica napus* is the second most abundant oilseed crop worldwide, contributing an estimated 12% of the world’s vegetable oil production [25], and this production is influenced by environmental stresses [26]. For example, seed survival and germination were reduced owing to heat stress or high temperatures, while low growth and development were reportedly caused by drought stress, which ultimately led to lower crop production. In previous studies, the POD gene family was involved in stress tolerance in different crops, including rice, maize, sorghum, and soybeans. Therefore, the present study selected the POD gene family for genome-wide identification in *B. napus*, using multi-omics analysis for the characterization, classification, structural diversification, genomic distribution, and identification of POD genes in response to multiple abiotic stresses.

## 2. Results

### 2.1. Characterization of BnPOD Gene Family 

A total of 109 genes were identified in the *B. napus* genome, using Arabidopsis POD proteins as query sequences after removing redundant sequences. All the identified POD proteins were renamed based on their physical position in the *B. napus* genome ZA11 viz. *BnAPOD01_BnCPOD109*. The predicted protein length and MW of the *POD* genes in *B. napus* varies from 279 aa/31.8118 kDa to 568 aa/62.91441 kDa. The theoretical pI ranged from 4.4 (*BnCPOD57*) to 10.11 (*BnAPOD13*), while GRAVY ranged from −0.355 (*BnAPOD18)* to 0.244 (*BnCPOD59*). Moreover, the in silico subcellular localization of all the *BnPOD*-predicted proteins revealed a wide range of cellular compartmentalization, including extracellular spaces, chloroplasts, and mitochondria (Table S1). Detailed information about the predicted protein length, pI, MW, chromosomal location, and other related information is shown in Table S1.

### 2.2. Phylogeny of POD Genes in B. napus

**Figure 1:**
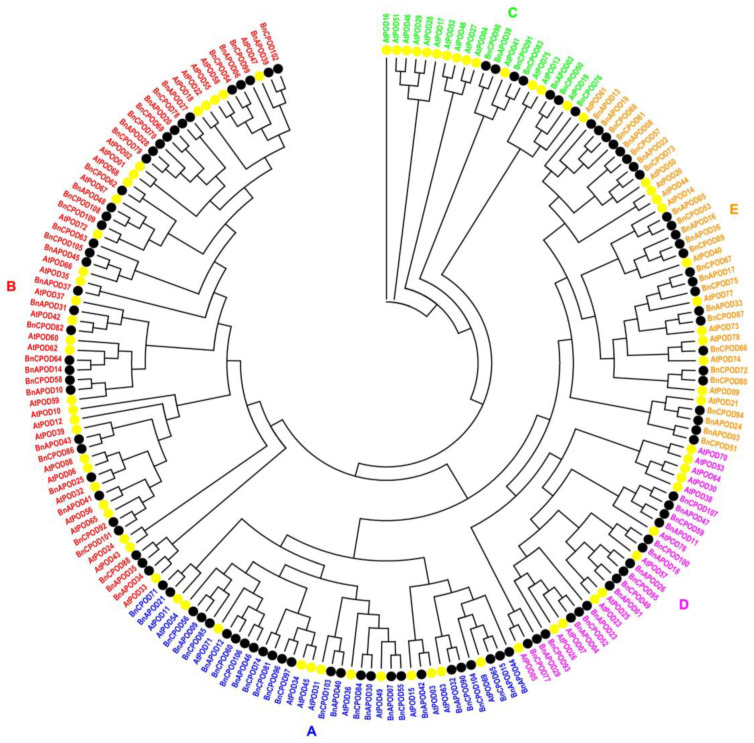
The phylogenetic tree of *B. napus* and *A. thaliana* POD proteins. The maximum likelihood phylogenetic tree was constructed using MEGA 11 with 1000 bootstrap replicates. The phylogenetic tree was clustered into 5 subclades (A–E). A distinct color represents each subclade.

To determine the homology and similarity among the BnPOD proteins and with those in Arabidopsis, a phylogenetic tree was generated with a bootstrap set at 1000 replicates. The phylogenetic analyses showed that all the *PODs* were clustered into five phylogenetic subclades, namely from A to E (Figure 1), based on the gene structure. The large subclades, A and B, had 62 (35 *BnPODs* and 27 *AtPODs*) and 38 (26 *BnPODs* and 12 *AtPODs*) *PODs*, respectively, whereas 37 and 28 *PODs* were found in subclades E and D, respectively. Similarly, 21 *PODs,* including 7 *BnPODs* and 14 *AtPODs*, were found in subclade C (Figure 1). These findings suggested the diversification of POD proteins in *B. napus*.

### 2.3. Gene Structural Analysis of BnPOD Proteins

**Figure 2:**
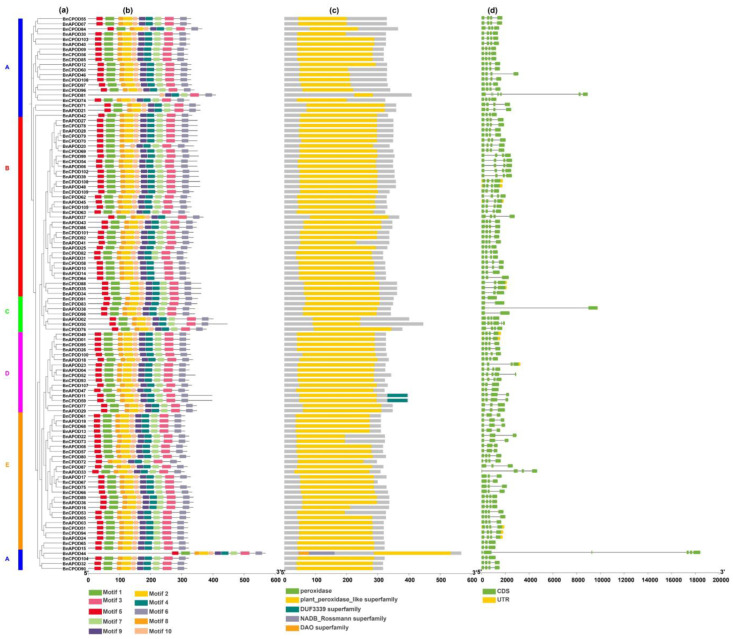
Gene structure of *POD* genes in *B. napus*. (**a**) The phylogenetic tree shows all the *BnPOD* genes in the five subclades. (**b**) Conserved motif analysis conducted using MEME Suite. A total of 10 motifs were predicted. (**c**) The domain organization of *BnPODs*. (**d**) Exon–intron organization of *BnPODs*. The A, B, C, D and E represent each subclades.

For this purpose, we used all the identified BnPOD genes for the detection of the exon–intron organization and conserved protein domains (Figure 2d). The exon–intron analysis revealed that the number of exons in *BnPODs* ranged from 2 to 7 among all the phylogenetic clades. For example, *BnCPOD81* had seven exons in clade A, while *BnAPOD14*, *BnAPOD15*, *BnCPOD57*, and *BnCPOD65* had three exons (Figure 2d). Similarly, *BnAPOD33* and *BnCPOD80* had four exons in clades B and E, whereas *BnAPOD38*, *BnCPOD83*, *BnCPOD91*, and *BnCPOD98* had two exons, but *BnCPOD50* had six exons in clade C. Generally, *POD* genes in the same subgroup show similar exon–intron features, providing evidence of their phylogenetic relationship (Figure 2d).

Furthermore, motif analysis was performed using the MEME database in accordance with the phylogenetic relationship. As a result, *BnPODs* that clustered in the same clade shared common motif compositions, indicating functional similarity among the members of the same *BnPOD* subclade. The InterProScan annotation of the MEME motif analyses revealed that eight motifs (motifs 1–4, 6, 7, 9, and 10) were noted as POD protein motifs, which are characteristic features of the peroxidase gene family. At least six motifs (1–4 and 7–10) were annotated as Heme peroxidases and were found in 95% of the sequences (Table 1 and Figure 2b). The *cis*-regulatory elements are involved in gene expression regulation. We used 2 kb long promoter sequences of all the *BnPODs* to scan the different types of regulatory elements, using the PlantCARE database. 

**Figure 3:**
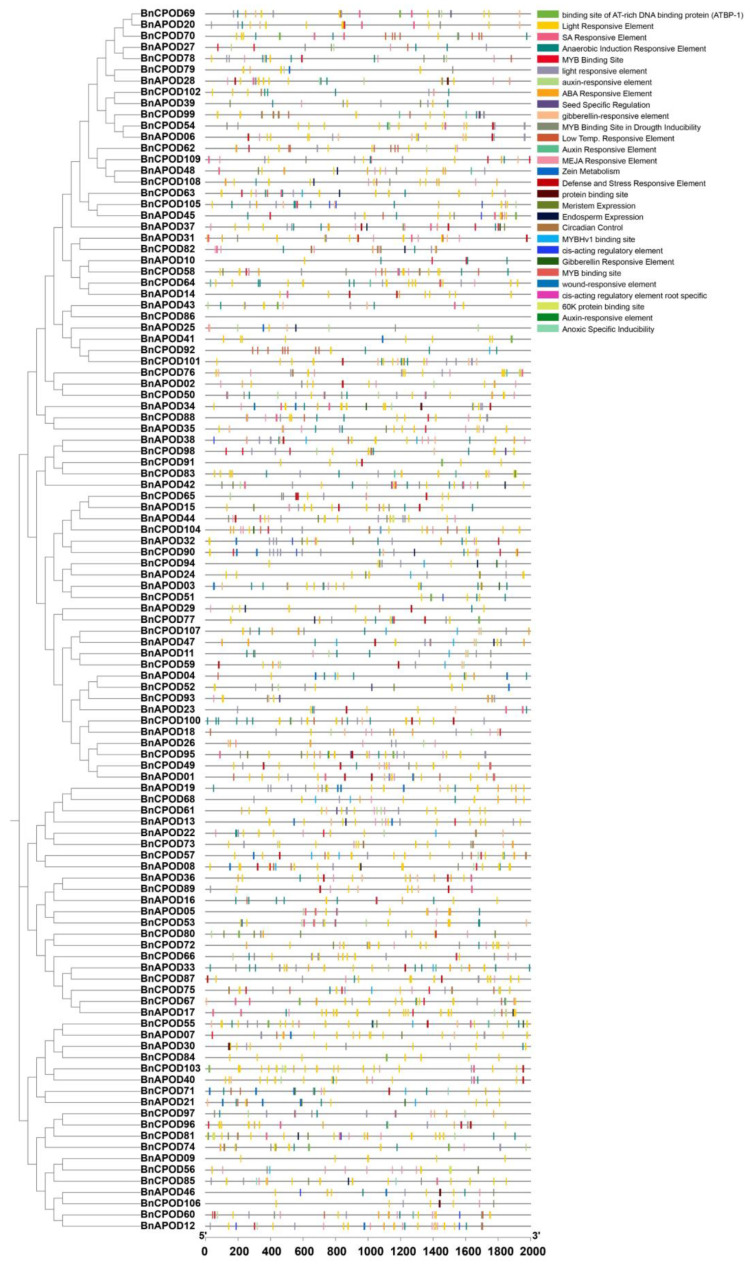
The analysis of the *BnPOD* promoter regions. The 2 kb sequences of the *BnPOD* gene-promoter regions were extracted from and analyzed using the PlantCARE database.

All the identified promoters were classified into two categories: (i) hormone-responsive and (ii) stress-responsive elements. Among the hormone-responsive elements, we identified ABRE (abscisic-acid-responsive element), the CGTCA/TGACG motif (methyl-jasmonate-responsive element), the TGA element (auxin-responsive element), and the TCA element (salicylic-acid-responsive element) (Figure 3 and Table S2). Similarly, among the stress-related responsive elements were LTR (low-temperature-responsive element), G-box (pathogen-inducible element/positive regulator of senescence), the WUN motif (wound-responsive element), MBS (MYB binding site in drought inducibility), TC-rich repeats (defense and stress responses), and STRE (stress-responsive element) (Figure 3 and Table S2). These findings indicate the diverse roles of class III peroxidase genes based on their gene structure.

### 2.4. Chromosomal Distribution and Duplication Events of POD Genes

**Figure 4:**
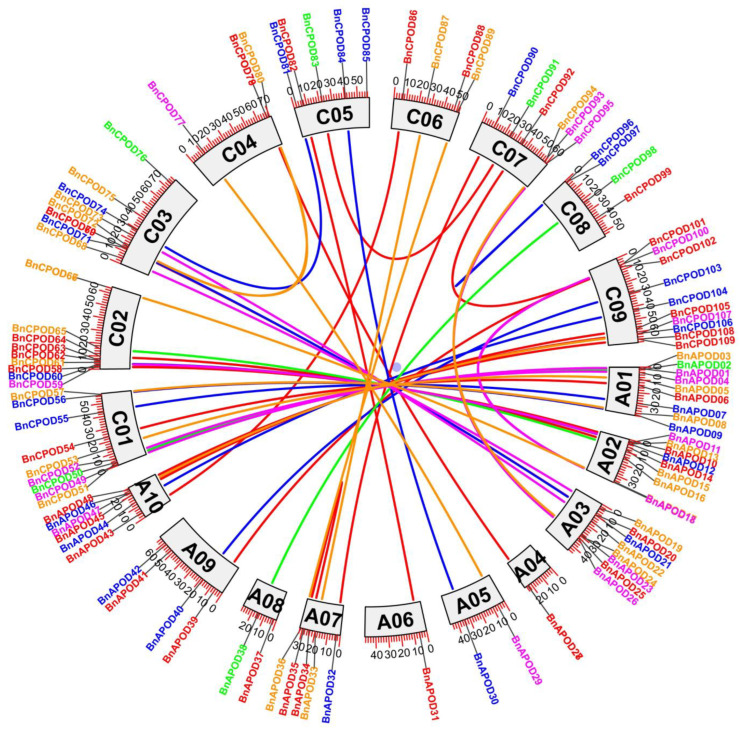
Circos plot of *POD* gene duplication in *B. napus*. The different colors represent the genes found in different (A–E subclades) subgroups, and the lines in the middle show segmental and tandem duplications between different chromosomes.

The *BnPOD* genes were unevenly distributed in all the chromosomes in the *B. napus* genome. For example, chromosomes C02 and C09 had 10 BnPODs; A01, A02, C01, and C03 had 9 BnPODs each; A10/C07 had 6 BnPODs; A07/C05 had 5 BnPODs; A09, C04, C06, and C08 had 4 BnPODs; A04, A05, and A08 had 2 BnPODs; and A06 had only 1 BnPOD (Table S1). Moreover, the expansion of a gene family occurs owing to the duplication events arising at the whole genome, and we identified duplicate gene pairs among the BnPODs based on sequence similarity. As a result, 44 duplicate events corresponding to 109 BnaPODs were identified with >80% sequence similarity. Gene pairs were selected as tandemly duplicated if the sequence was found within a 100 kb window of the duplicated genomic region. According to these criteria, the *BnAPOD35*–*BnAPOD34* and *BnCPOD97*–*BnCPOD96* gene pairs were identified as tandemly duplicated (Table S3 and Figure 4). The remaining 43 BnPODs, including *BnCPOD55*–*BnAPOD07*, *BnCPOD84*–*BnAPOD30*, *BnCPOD100*–*BnAPOD18*, *BnAPOD04*–*BnCPOD52*, and *BnCPOD107*–*BnAPOD47*, were identified as segmentally duplicated (Table S3). C09, A01, A02, A03, A10, C01, C02, and C03 had undergone the highest number of segmental duplication events, indicating their key contribution to the expansion of the *BnPOD* family in *B. napus* (Figure 4). 

**Figure 5:**
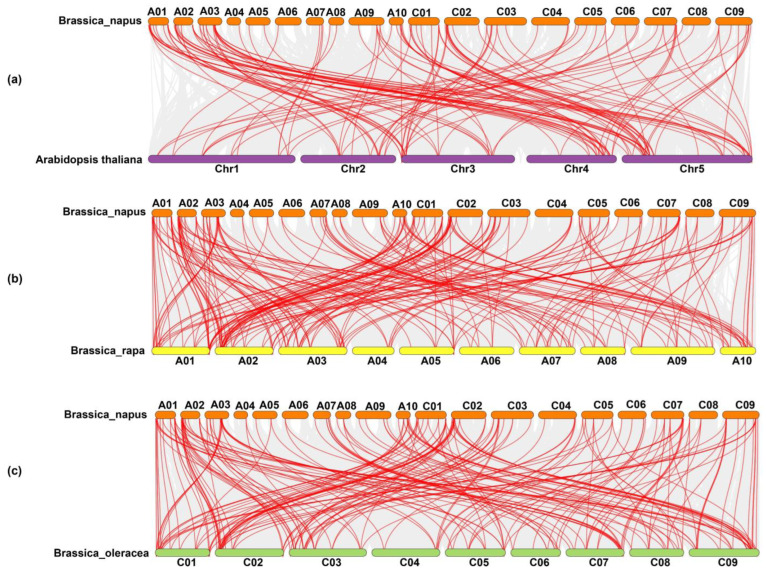
Dual synteny plots between (**a**) *B. napus* and *A. thaliana*, (**b**) *B. napus* and *B. rapa*, and (**c**) *B. napus* and *B. oleracea* genomes, with orthologous *POD* genes shown with red connecting lines.

The Ka, Ks, and Ka/Ks values are key indicators of selection pressures. In general, a Ka/Ks ratio of >1 refers to positive selection; a Ka/Ks ratio of <1 refers to purifying selection, and a Ka/Ks ratio equal to 1 refers to neutral selection. The Ka/Ks ratio varies between 0.05 and 0.65, indicating that all the *BnPOD* gene pairs exhibited negative or purifying selection (Ka/Ks < 1) (Table S3). These findings demonstrated that the purifying or negative selection was involved in maintaining the conservation of the *BnPOD* gene structure during the domestication or evolution process. Further, we estimated the divergence time between duplication events, and the results showed that the estimated divergence time ranged from 0.86 to 32.67 million years ago (MYA), with an average of ~6.15 MYA (Table S3). The syntenic relationships between different chromosome segments of different species provide a source of information about the origin of gene family members. For this purpose, we performed synteny analysis between *POD* genes from the *B. napus*, *B. rapa*, *B. oleracea*, and *Arabidopsis* genomes (Table S4 and Figure 5). Our results revealed that 55 *BnPODs* were placed in 106 colinear blocks with *B. oleracea*, 49 *BnPODs* in 102 colinear blocks in *B. rapa*, and 94 *BnPODs* in 127 colinear blocks in the *Arabidopsis* genome (Table S4 and Figure 5). 

### 2.5. miRNA-Mediated BnPOD Regulation

**Figure 6:**
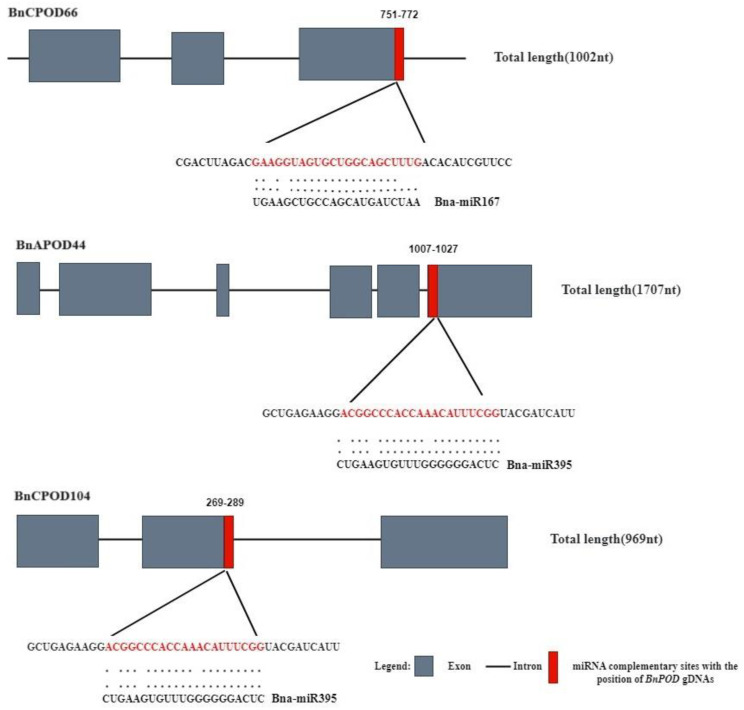
A network illustration of the regulatory associations among the putative miRNAs and *BnPOD* genes. The red color indicates the miRNA complimentary site with the position of *BnPODs* gDNAs.

In recent years, various studies have unveiled the importance of miRNA-mediated gene expression regulation in response to biotic and abiotic stresses. In this study, we identified two putative miRNAs targeting three *BnPOD* genes. The results showed that members of the Bn-miR167 family targeted *BnCPOD66* and members of the Bn-miR395 family targeted *BnAPOD44* and *BnCPOD104* (Table S5 and Figure 6). 

### 2.6. Expression Profiling of BnPOD Genes in Various Tissues

**Figure 7:**
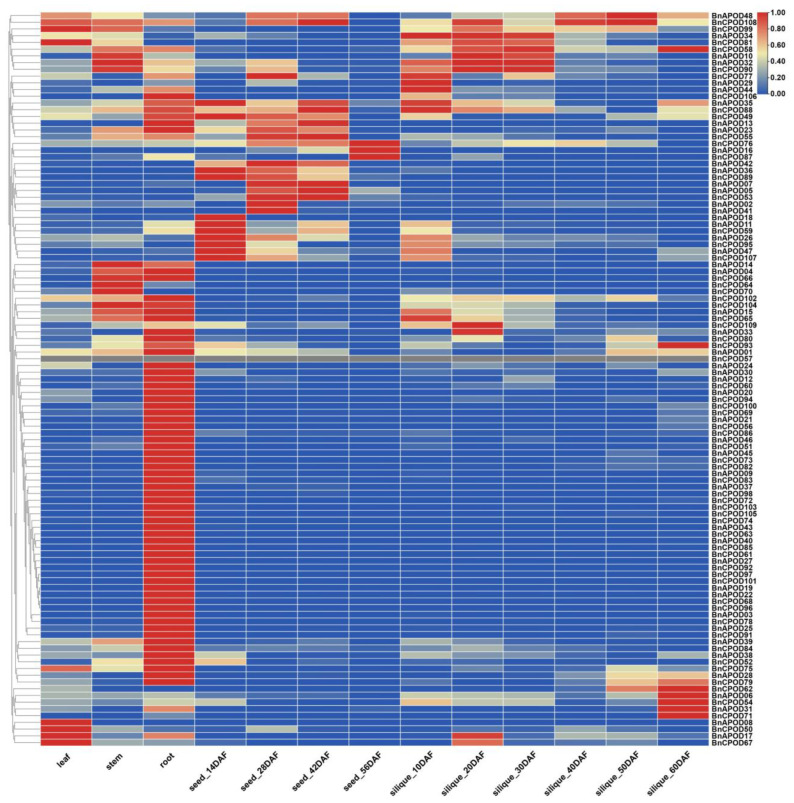
Tissue/organ-specific expression patterns of *BnPOD* genes in *B. napus* according to in silico RNA-seq data. DAF; days after flowering.

The expression patterns of the *BnPODs* genes in different tissues, including the leaves, stems, roots, and seeds at 14 DAF (days after flowering), 28 DAF, 42 DAF, and 56 DAF and the siliquae at 10 DAF, 20 DAF, 30 DAF, 40 DAF, 50 DAF, and 60 DAF, were investigated. The analysis of the RNA-seq revealed spatial expression patterns of *BnPODs* in different tissues. For instance, most genes were expressed in roots compared with other tissues. The expressions of *BnCPOD50*, *BnAPOD17*, and *BnCPOD87* were confined to leaves, but *BnCPOD62, BnAPOD06, BnCPOD51*, *BnAPOD31*, and *BnCPOD71* were expressed only in 60 DAF siliquae. The expressions of *BnAPOD18*, *BnAPOD11*, *BnCPOD59*, *BnAPOD26*, *BnCPOD95*, *BnAPOD47*, and *BnCPOD107* were higher in seeds at 14 DAF, while *BnCPOD76*, *BnAPOD16*, and *BnCPOD87* were expressed in 56 DAF seeds (Figure 7). This temporal expression pattern indicated that the dynamic roles of the candidate *BnPODs* might play an important role in the growth and developmental processes of *B. napus*.

### 2.7. Expression Patterns of BnPODs during Abiotic Stresses

**Figure 8:**
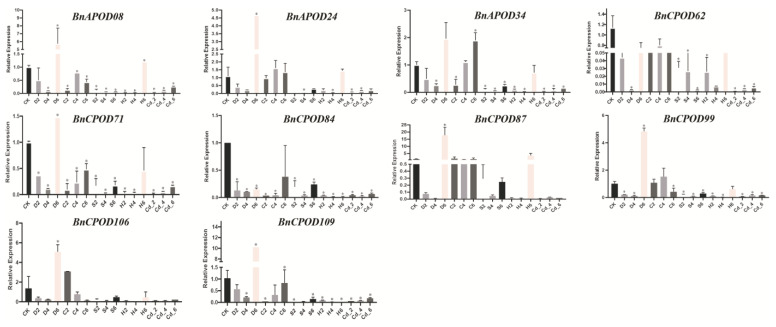
Expression patterns of selected *BnPODs* subjected to different abiotic stresses. The data are presented with ±standard errors. Statistically significant differences are denoted by asterisks * *p* ≤ 0.05. CK, control; D, drought; C, cold; S, salt; H, heat; Cd, cadmium; numbers 2, 4, and 6 indicate time intervals of 2 h, 4 h, and 6 h, respectively. The samples at 0 h were used as a control (CK).

We selected POD genes according to the RNA-seq expression profile; for example, *BnAPOD24*, *BnAPOD34*, and *BnCPOD99* were highly expressed in all the tissues; *BnCPOD62* and *BnCPOD84* were expressed in partial tissues, such as roots, leaves, stems, and siliquae; and *BnAPOD08* was highly expressed in leaf tissues. To investigate the expressions of the above *BnPODs* in *B. napus* during abiotic stresses (salinity, drought, heat, cold, and cadmium chloride), these tissues were analyzed at 0 h, 2 h, 4 h, and 6 h. With RT-qPCR, the expressions of ten selected *BnPODs* were examined under the applied abiotic stresses. The expression patterns of all the *BnPODs* were significantly changed under the applied stresses. For instance, under drought stress, all the *BnPODs* were initially downregulated at 2 h and 4 h but significantly upregulated at 6 h except for *BnCPOD62* and *BnCPOD84*. Under cold stress, only *BnAPOD34* was upregulated at 6 h, but *BnAPOD24* and *BnCPOD99* were upregulated at 4 h. Although *BnCPOD87* and *BnCPOD106* were upregulated at 2 h, they were downregulated in other time intervals. Likewise, under heat stress, the expressions of *BnAPOD08*, *BnAPOD24*, and *BnCPOD87* were upregulated at 6 h. However, it is interesting that all the selected genes were downregulated under the salt and heavy-metal stresses (Figure 8). Our results indicate that PODs might play a pivotal role in tolerance to applied abiotic stresses.

## 3. Discussion

*Brassica napus* is an allotetraploid crop and is susceptible to environmental stresses, including cold, heat, salt, drought, and heavy metals [27], which negatively influence the growth and final production [28]. Class III peroxidases (PODs) play critical roles in regulating plant physiology, particularly responses to abiotic stresses [24]. Consequently, the systematic genome-wide characterization of the *POD* genes in different crops is essential to clarify their biological roles in plants, particularly in growth and defense responses. The *POD* gene family has been extensively studied in different plant species, such as *A. thaliana* [17], *Saccharum officinarum* [29], *O. sativa* [7], *N. tabacum* [30], *Z. mays* [16], *Betula pendula* [31], *P. bretschneideri* [19], and *M. esculenta* [18], while the comprehensive identification of *PODs* in *B. napus* is still lacking.

In this study, we identified a total of 109 *POD* genes in *B. napus,* which is more than those reported in *A. thaliana* (73) [17] and *M. esculenta* (91) [18] but fewer than those in *O. sativa* (138) [7] and *G. max* (124) [22]. This higher number of *POD* genes in *B. napus* might be owing to its allopolyploid nature. We first analyzed the physical locations of the *BnPODs* in *B. napus* throughout the A and C genomes. We found that all the *BnPODs* are unevenly distributed in all the chromosomes, which is consistent with their chromosomal distributions in *A. thaliana* [17], *S. officinarum* [29], *O. sativa* [7], *N. tabacum* [30], and *Z. mays* [16]. Gene duplication, such as segmental duplication, tandem duplication, and whole-genome duplication, is responsible for the evolution and expansion of the gene family [18]. Next, 44 paralogous *POD* genes were identified in *B. napus*, indicating that segmental duplication contributed to the *BnPOD* expansion. Accumulated evidence has demonstrated that duplication events have been important for gene expansion in the *POD* family. The results of our study are consistent with *POD* expansion in *O. sativa* [7], *P. pyrifolia* [19], *G. max* [22], and *M. esculenta* [18]. 

Various *cis*-regulatory elements have been identified and can regulate the expression of stress-related genes [32]. We found multiple *cis*-acting elements in the upstream regions of the *BnPOD* family, including hormone-responsive and stress-responsive elements. Similar findings have also been reported for *V. vinifera POD* genes [21]. Antioxidant genes, such as *SOD*, *CAT*, and *APX*, regulate the underlying stress-tolerant mechanisms [33,34,35]. For instance, *PODs* significantly play an important role in mitigating different abiotic stresses [11,12,36]. In this study, we selected a few *BnPOD* genes for various abiotic stresses, which are located on ChrA02, A06, C02, C03, C04, C05, and C09. The phylogeny analysis of the selected genes revealed that *BnAPOD08* and *BnCPOD87* belong to subclade E; *BnAPOD34*, *BnCPOD62*, and *BnCPOD109* belong to subclade B; and *BnCPOD71*, *BnCPOD84*, *BnCPOD99*, and *BnCPOD106* belong to subclade A (Figure 2). 

Notably, *BnPOD* genes showed large variations in their expression profile patterns, revealing their roles in different stress responses and defense responses in *B. napus*. Meanwhile, individual *POD* genes are usually sensitive to one specific external stress, and few genes are widely responsive to various biotic and abiotic stresses. For example, all the selected *BnPOD* genes are responsive to all the applied stresses, including cold, drought, salt, and heavy metals. Previous studies have suggested that the overexpression of *POD* genes, such as *AtPrx22*, *AtPrx39*, and *AtPrx69*, in *A. thaliana* is involved in cold tolerance [37]; also, *POD* family genes play a role in stresses in *Populus* and *G. max* [22,38] under drought, heat [39], salinity, and cadmium stresses. In summary, the expression patterns of the *BnPODs* under various abiotic stresses are complex and diverse and may be related to the functional diversity of the *POD* gene family members. These results provide useful insights into the potential capabilities of *BnPODs* under various abiotic stresses.

## 4. Materials and Methods

### 4.1. Mining of BnPOD Family in B. napus

POD protein sequences of *A. thaliana* were retrieved from the TAIR database (https://www.arabidopsis.org/, accessed on 2 August 2023) [40] and used as a query sequence in the *B. napus* genome, using the BlastP tool (https://blast.ncbi.nlm.nih.gov/Blast.cgi, accessed on 2 August 2023). After the removal of duplications, the non-redundant protein sequences were subjected to SMART (http://smart.emblheidelberg.de/, accessed on 2 August 2023) and NCBI conserved-domain searches (https://www.ncbi.nlm.nih.gov/Structure/cdd/wrpsb.cgi, accessed on 4 August 2023). The final POD proteins were renamed according to their physical positions in the *B. napus* genome. Furthermore, the physicochemical properties of the BnPOD proteins, including the protein length (aa), theoretical isoelectric point (pI), molecular weight (kilodaltons; kDa), and grand average of hydropathicity (GRAVY) were computed using ProtParam (http://web.expasy.org/protparam/, accessed on 6 August 2023). WoLF PSORT (https://wolfpsort.hgc.jp/, accessed on 9 August 2023) was used to predict the in silico subcellular localizations of all the deduced POD proteins [41].

### 4.2. Phylogeny of B. napus POD Proteins

To draw the phylogenetic tree of the POD proteins, the protein sequences of *B. napus* and *A. thaliana* were first aligned using MEGA software (Version 11) [42], and the maximum likelihood tree was then generated using iq-tree software (http://www.iqtree.org, accessed on 14 August 2023). The bootstrap values were set at 1000 replicates. The generated phylogeny was further modified using ITOL (https://itol.embl.de/, accessed on 15 August 2023) [43]. 

### 4.3. Gene Structural Analysis of B. napus POD Proteins

The intron and exon organizations within the *BnPOD* genes were predicted using TBtools (version 2.003) [44]. For the identification of the conserved motifs within the BnPODs, we used MEME Suite (https://meme-suite.org, accessed on 19 August 2023) [45], with the number of motifs set at 10.

For the identification of the *cis*-regulatory elements within the promoters of the *BnPOD* genes, a 2 kb sequence upstream from the start codons was submitted to the PlantCARE website (http://bioinformatics.psb.ugent.be/webtools/plantcare/html/, accessed on 25 August 2023) [46].

### 4.4. Gene Duplication and Evolutionary Analysis of BnPOD 

We analyzed the duplication events of the BnaPOD gene family using MCScanX (https://github.com/wyp1125/MCScanX, accessed on 26 August 2023), and visualized them in Circos, as described by Waseem et al. [47]. Furthermore, the Ka and Ks substitutions between the gene pairs were also calculated as described by Aslam et al. [48]. The divergence time (T, MYA; millions of years ago) was calculated as follows: T = Ks/2R (where R = 1.5 × 10^−8^ and is synonymous with the number of substitutions per site per year) [49]. Moreover, the syntenic relationship among *B. napus*, *B. rapa*, *B. oleracea*, and *Arabidopsis thaliana* was assessed using MCScanX and visualized in TBtools. 

### 4.5. Prediction of Putative miRNAs Targeting BnPOD Genes 

To predict the potential targets of *BnPOD* genes by miRNAs, the psRNATarget database (http://plantgrn.noble.org/psRNATarget/, accessed on 27 August 2023) [50] was employed with default parameters, including the number of expectations being set at 3. The interaction network between the predicted miRNAs and their corresponding *BnPOD* genes was visualized in Cytoscape (version 3.10.1) [51,52].

### 4.6. Expression Analysis of BnPOD Genes in Different Tissues

The expression data of the *BnPOD* genes were retrieved from the BnPIR database (http://cbi.hzau.edu.cn/bnapus/index.php, accessed on 3 September 2023) [53]. We evaluated the temporal and spatial expression data of the 109 *BnPOD* genes using RNA-seq data for various *B. napus* tissues, including the roots, stems, leaves, seeds, and siliquae [34], as well as at different developmental stages. 

### 4.7. Plant Materials and Abiotic Stresses

We conducted a controlled laboratory experiment to investigate the *BnPOD* gene responses under various abiotic stresses. Plants of *B. napus* var. 383-5 were grown in a hydroponic solution, using the full Hoagland solution (2.5 mM Ca(NO_3_)_2_, 2 mM KCl, 1 mM MgSO_4_, 0.5 mM NH_4_H_2_PO_4_, 0.5 mM CaCl_2_, 25 µM H_3_BO_3_, 5 µM MnSO_4_, 0.8 µM ZnSO_4_, 0.3 µM CuSO_4_, 0.1 µM (NH_4_)_6_Mo_7_O_24_, and 100 µM EDTA, 2NaFe), with continuous aeration [54]. Other experimental conditions, including a 14 h light and 10 h dark cycle, were maintained. Subsequently, the plants were exposed to various abiotic stresses, including NaCl (150 mM), CdCl_2_ (cadmium chloride, 2.5 mM), cold stress at 8 °C, heat stress at 42 °C, and drought stress. For the drought stress, the plants were subjected to a drought stress with a 15% PEG-6000 solution. Each stress treatment was carried out with three independent biological replicates. The samples were collected at 0 h, 2 h, 4 h, and 6 h. All the samples were immediately frozen in liquid nitrogen and stored at −80 °C for the subsequent analysis.

### 4.8. RNA Isolation and Real-Time Quantitative PCR Expression Analysis

The total RNA was extracted from the collected samples, using an RNAprep Pure Plant Kit (Lot no. Q5510, Tiangen, Beijing, China) following the manufacturer’s protocol. The concentration of the extracted RNA samples was measured using a NanoDrop (2000 C spectrophotometer from Thermo Fisher Scientific, Waltham, MA, USA). DNase I treatment was used to remove the genomic DNA [55]. The first complementary strand of DNA (cDNA) was synthesized using HiScript 1st strand cDNA synthesis kit (V22.1, Vazyme Biotech Co., Ltd., Sanya, China). The cDNA of samples was assessed by RT-qPCR using ChamQTM SYBR RT-qPCR Master Mix (Vazyme Biotech Co., Ltd., Sanya, China). The expression level of the *Actin* was used as internal control. The RT-qPCR was performed with three independent biological replicates. The relative expression level of *BnPODs* was calculated using the 2^−ΔΔCT^ method [56]. The primers used in this study are listed in Table S6.

## 5. Conclusions

This research provides the first comprehensive genome-wide analysis of the *POD* family genes in *B. napus*. We identified 109 *POD* family genes from the BnPIR database of *B. napus*. Then, we studied their basic classification, gene structure, motif distribution and duplication events. Additionally, RT-qPCR analysis revealed that the selected *BnPOD* genes were significantly expressed under abiotic stresses, suggesting that these genes can be potential candidates for *B. napus* crop improvement. In the future, attempts to increase the stress tolerance of *B. napus* will require knowledge of the specific peroxidases that are up-regulated by stresses. In summary, our discoveries offer valuable perspectives into the functional dimensions of *POD* family genes, which open the way for upcoming investigations in economically significant *B. napus* crops.

## Figures and Tables

**Table 1:** Sequences of ten predicted MEME motifs with InterProScan annotations.

Motif	Sequence	InterProScan Annotation
Motif-1	DPRJAAALLRLHFHDCFVNGCDASVLLDS	Heme peroxidases
Motif-2	AACPGVVSCADILALAARDSVVLAGGPSW	Heme peroxidases
Motif-3	DPGTPNTFDNSYFKNLRQGKGLLQSDQAL	Heme peroxidases
Motif-4	KDLVALSGAHTIGFAHCGSFTBRL	Heme peroxidases
Motif-5	AQLSPGFYDKSCPNAESIVRN	-
Motif-6	FFRAFAKAMVKMGNIGVLTGSQ	Heme peroxidases
Motif-7	GDPDPTLBPTYAAQLRKKCPR	Heme peroxidases
Motif-8	SLRGFEVIDDIKAALE	-
Motif-9	PSPFDNVSQLITKFAAKGLNV	Heme peroxidases
Motif-10	VPLGRRDGRVSNASE	Heme peroxidases

## Data Availability

The authors confirm that the data supporting the findings of this study are available within the article and its Supplementary Materials.

## Supplementary Materials

The following supporting information can be downloaded at https://www.mdpi.com/article/10.3390/plants13070942/s1: Table S1: Characteristics of PODs in *Brassica napus*; Table S2: Identification of different *cis*-regulatory elements in promoters of *BnaPODs*; Table S3: Parameters related to gene duplication and evolution of *BnPODs* in *B. napus*; Table S4: Collinearity analysis among *Brassica napus*, *Brassica rapa*, *Brassica oleracea*, and *Arabidopsis thaliana*; Table S5: *BnaPOD*–miRNA prediction using psRNATarget server; Table S6: List of all the primers used in this study.
